# The Role of Human Papilloma Virus in Dictating Outcomes in Head and Neck Squamous Cell Carcinoma

**DOI:** 10.3389/fmolb.2021.677900

**Published:** 2021-06-23

**Authors:** Shane Brennan, Anne-Marie Baird, Esther O’Regan, Orla Sheils

**Affiliations:** ^1^ School of Medicine, Faculty of Health Sciences, Trinity College, Dublin, Ireland; ^2^ Department of Histopathology, St. James’s Hospital, Dublin, Ireland

**Keywords:** head and neck cancer, oropharyngeal cancer, cancer recurrence and metastasis, HPV—human papillomavirus, HPV genotype, somatic mutation analysis

## Abstract

The Human Papilloma Virus (HPV) is an oncogenic virus which is associated with the development of head and neck squamous cell carcinoma (HNSCC), predominantly within the oropharynx. Approximately 25% of oropharyngeal squamous cell carcinoma (OPSCC) cases worldwide are attributable to HPV infection, with an estimated 65% in the United States. Transmission is *via* exposure during sexual contact, with distinctive anatomical features of the tonsils providing this organ with a predilection for infection by HPV. No premalignant lesion is identifiable on clinical examination, thus no comparative histological features to denote the stages of carcinogenesis for HPV driven HNSCC are identifiable. This is in contrast to HPV-driven cervical carcinoma, making screening a challenge for the head and neck region. However, HPV proffers a favorable prognosis in the head and neck region, with better overall survival rates in contrast to its HPV negative counterparts. This has resulted in extensive research into de-intensifying therapies aiming to minimize the morbidity induced by standard concurrent chemo-radiotherapy without compromising efficacy. Despite the favorable prognosis, cases of recurrence and/or metastasis of HPV positive HNSCC do occur, and are linked with poor outcomes. HPV 16 is the most frequent genotype identified in HNSCC, yet there is limited research to date studying the impact of other HPV genotype with respect to overall survival. A similar situation pertains to genetic aberrations associated in those with HPV positive HNSCC who recur, with only four published studies to date. Somatic mutations in TSC2, BRIP1, NBN, TACC3, NFE2l2, STK11, HRAS, PIK3R1, TP63, and FAT1 have been identified in recurrent HPV positive OPSCC. Finding alternative therapeutic strategies for this young cohort may depend on upfront identification of HPV genotypes and mutations which are linked with worse outcomes, thus ensuring appropriate stratification of treatment regimens.

## Introduction

Oncogenic Human Papilloma Virus (HPV) is associated with the development of squamous cell carcinoma of the anogenital (cervical, vaginal, vulvar, penile, and anus) and the head and neck region (Bansal et al., 2016). HPV positive head and neck squamous cell carcinoma (HNSCC) are most commonly found within the oropharynx (Elrefaey et al., 2014). The oropharynx consists of the base of tongue, soft palate, uvula, lateral and posterior pharyngeal wall, tonsils, tonsillar fossa and tonsillar pillars (Amin MB et al., 2017). Further analysis has illustrated that carcinogenesis due to HPV within the oropharynx is most prominent in the tonsil and base of tongue (Haeggblom et al., 2017; Marklund et al., 2020). The global incidence of HPV positive OPSCC is increasing with figures estimating that 25% of OPSCC cases worldwide are attributable to HPV infection, in contrast to North America which has a higher prevalence of approximately 65% (WHO, 2014; Stein et al., 2015).

Early studies on HPV associated cancer arose from work on cervical squamous cell carcinomas (Bosch et al., 2002). However, the pathogenesis for HPV driven OPSCC does not mirror that of the cervix. Inter-site discrepancies exist, including HPV genotype prevalence and associated genomic alterations (Cancer Genome Atlas Network, 2015; The Cancer Genome Atlas R, 2017; LeConte et al., 2018).

When stratified into HPV positive and negative OPSCCs, it has been identified that HPV positive OPSCCs have a distinctive mutational landscape compared with HPV negative counterparts (Gillison et al., 2000). Thus, although HPV is a risk factor for the development of OPSCC, it is associated with a favorable overall prognosis (Hennessey et al., 2009; Economopoulou et al., 2020). Despite this, 15% of patients with HPV positive OPSCC progress to recurrence or local and/or distant metastasis (Masterson et al., 2014; Gleber-Netto et al., 2019).

The purpose of this article is to review HPV carcinogenesis within the head and neck region, focusing on pathological and molecular discrepancies in contrast to the cervical region. In view of the favorable prognosis for HPV positive OPSCC, studies have primarily focused on discerning a tailored therapeutic strategy for HPV positive OPSCC to minimize long-term side-effects derived from intensive chemotherapy, radiation therapy (RT) and/or surgical intervention (Strohl et al., 2020). There is a limited field of knowledge at present pertaining to the HPV genotypes and DNA mutations identified in those with HPV positive OPSCC who develop local recurrence and/or distant metastasis. This review appraises the literature surrounding the HPV genotypes and genetic aberrations associated with HPV positive OPSCC with disease progression. De-intensification strategies would be deleterious for this cohort of patients, and thus research should focus on identifying individuals at risk of recurrence and/or metastasis. Thus, the aim of this review article is to illustrate that further research is required to address this group of patients who have a clinical unmet need.

## Human Papilloma Virus Within the Head and Neck Region

### Human Papilloma Virus Transmission

This epitheliotropic oncogenic virus is transmitted *via* skin-to-skin contact, skin-to-mucosa contact, or mucosa-to-mucosa contact. HPV transmission is thus predominantly *via* sexual behaviors including oral sex, vaginal sex and oral-anal contact (D'Souza et al., 2007). The changing dynamic of increased sexual partners and changes in common practices of sexual behavior, is believed to have led to the increased prevalence of HPV and thus the increasing incidence of HPV driven OPSCC (D'Souza et al., 2007). The risk of contracting multiple HPV genotypes is directly correlated to the lifetime number of sexual partners (Vaccarella et al., 2006). A large-scale multi-center study conducted by INHANCE reported an increased risk of base of tongue and tonsillar cancer for individuals with a history of greater than five sexual partners, greater than three oral sexual partners and an earlier age at sexual debut (Heck et al., 2010). In addition, a two-fold increased risk with one to five lifetime oral sexual partners and a fivefold increase if greater than seven oral sexual partners has been shown (Marur et al., 2010). Despite these compelling results, other studies have reported that between 8 and 40% of individuals diagnosed with HPV positive HNSCC reported never having oral sexual contacts (Chaturvedi et al., 2008; Marur et al., 2010). Further supporting this, a study in 2013 described no ‘significant’ correlation between oral HPV infection and oral sexual contact (Kreimer et al., 2013; Shigeishi and Sugiyama, 2016). In conclusion, the exact mechanism by which contraction occurs has yet to be elucidated.

### Predilection for the Oropharynx

Within the oropharynx, the tonsillar crypts of the palatine and lingual tonsils are the most frequent site for HPV positive OPSCC (Perry, 1994; Pai and Westra, 2009; Gelwan et al., 2017; Pan et al., 2018). The exact reasoning behind this predilection has not been clarified. Within the uterine cervix, it is theorized that HPV transmission is facilitated by micro-abrasions of the squamous lining mucosa with exposure of the underlying basement membrane. This permits entry of the virus to the basal region squamous epithelium, where stem cells are localized (Pai and Westra, 2009; Johnson et al., 2020). The perceived mechanism for transmission in the head and neck region is thought to be different, and it is the specific anatomical and histologic structure of the tonsil that deems it to be the favored site for HPV infection.

Firstly, the tonsils are composed of extensive tonsillar crypts, which increase the mucosal surface area. Secondly, the tonsillar surface is lined with non-keratinizing squamous epithelium, but within the crypts it is lined by reticulated squamous epithelium. This reticulated squamous epithelium has an intervening desmosomal network providing a scaffold-like structure linking the individual cells, thus making the layer penetrable and porous (Perry, 1994; Roberts et al., 2019). This provides a mechanism for HPV to migrate to the basal layer of the reticulated epithelium (Perry, 1994; Pai and Westra, 2009; Gelwan et al., 2017; Roberts et al., 2019).

A further structural component distinct to the tonsil is an anomaly of the basement membrane. Underlying most epithelial structures exists a continuous basement membrane as a further protective barrier. The tonsillar basal epithelial layer is separated from the underlying lymphoid tissue of the tonsil by a discontinuous basement membrane, facilitating viral transmission (Pai and Westra, 2009).

In addition, the oncogenic virus has the ability to evade the immune system response at this site (Johnson et al., 2020). Programmed Death Ligand 1 (PDL1) acts as a checkpoint regulator ensuring control of the adaptive immune system (Lyford-Pike et al., 2013a). It may be perceived unusual that a lymphoid organ, such as the tonsil would be a site for viral entry. However, PDL1 expression by squamous epithelial cells within the tonsillar crypts is elevated, in contrast to those on the surface (Lyford-Pike et al., 2013a). PDL1 interacts with Programmed Death 1 (PD1) receptors on T-lymphocytes with resultant inhibition of its activity. The downstream effect is the evasion of the immune response (Thunnissen et al., 2019). In conclusion, elevated PDL1 levels at the tonsillar crypts lead to reduced lymphocytic activity, permitting virus entry (Lyford-Pike et al., 2013b; Roberts et al., 2019; Wang et al., 2019).

### Human Papilloma Virus Life Cycle: Cervix Versus Head and Neck Region

**FIGURE 1:**
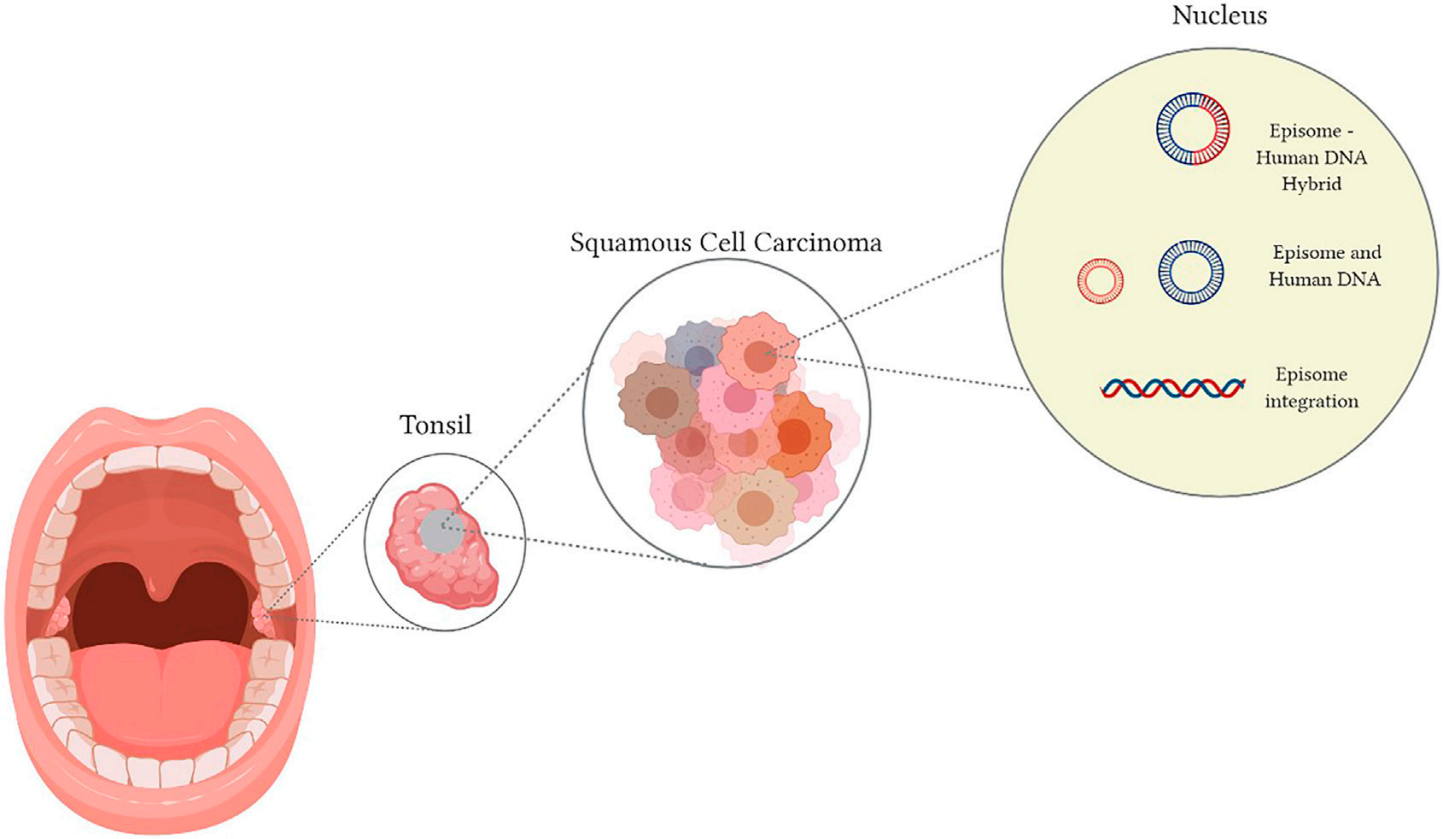
Human Papilloma Virus transmission into the host nucleus can result in **(A)** Viral DNA integration, **(B)** a hybrid of conjoined viral-DNA and host DNA, **(C)** independent viral-DNA, not integrated or linked with the host DNA.

Once transmission occurs, based on modelling from virus entry into cervical squamous epithelium, the virus integrates into the host DNA with resultant dysregulation of E1 and E2 expression (Roberts et al., 2019). This results in the upregulation of the E6 and E7 proteins (Lehoux et al., 2009; Balasubramaniam et al., 2019; Maglennon and Doorbar, 2012). Although it is known that HPV-associated cancers contain integrated viral-DNA, viral-DNA inclusion into the host genome is not essential or universally observed amongst all HPV related cancers (Kristiansen et al., 1994; McBride and Warburton, 2017; Gao et al., 2014). Viral-DNA can exist in episomal form within the cell or mixed episomal and integrated, referred to as viral-human hybrid episome. This mechanism is illustrated in Figure 1 (Gao et al., 2014; Olthof et al., 2014; Morgan et al., 2017; Roberts et al., 2019). If present in this extrachromosomal/episomal form, the viral DNA will acquire genetic aberrations resulting in dysregulated oncoprotein gene expression (McBride and Warburton, 2017). The prevalence of episomes that are not integrated into the host genome is far greater in HPV positive OPSCC than cervical squamous cell carcinomas (McBride and Warburton, 2017; Roberts et al., 2019). The implications pertaining to each of the various viral DNA constructs in the host have yet to be clarified.

### Pathogenesis of Squamous Cell Carcinomas: Cervix Versus Head and Neck Region

A well delineated stepwise progression from initial infection with HPV, to the development of premalignant dysplastic changes with resultant invasive squamous cell carcinoma of the cervix, is accompanied by gross features identifiable during colposcopy and distinctive cytological and histological findings (Maglennon and Doorbar, 2012). Infection with the oncogenic virus leads to evolving dysplastic changes, resulting in characteristic histological features classified by the percentage of epithelial layer involvement. Cervical intra-epithelial neoplasia (CIN) 1/low grade squamous intra-epithelial lesion (LSIL) denotes lower third basal layer displaying dysplastic changes. CIN 2 and CIN 3 are collectively referred to as high grade squamous intra-epithelial lesion (HSIL), showing two thirds, or full thickness dysplasia (Waxman et al., 2012). The dysplastic findings identified on microscopy correlate to the level of E6 and E7 protein expression (Doorbar et al., 2015). For example, in CIN 1, high levels of E6 and E7 expression are noted in the lower third of the squamous epithelium thickness. Above this point, in the upper two thirds, E1, E2 and E4 mRNA expression has been identified (Maglennon and Doorbar, 2012; Doorbar et al., 2015; Balasubramaniam et al., 2019). Genetic alterations resulting in the inactivation of the tumour suppressor gene cyclin dependent kinase inhibitor (CDKN2A), which has a key role in regulating cell cycle progression, has been identified in CIN 1 (Balasubramaniam et al., 2019). Further upregulation of Cyclin B1, budding uninhibited by benzimidazoles 1 homolog beta (yeast) (BUB1B) and minichromosome maintenance (MCM), all involved in mitosis and DNA replication, has been identified in CIN 2/3 (Balasubramaniam et al., 2019; den Boon et al., 2015). Upregulation of Phosphatidylinositol-4,5-Bisphosphate 3-Kinase Catalytic Subunit Alpha (PIK3CA) has been elicited in a minority of CIN 3 and a high proportion of invasive carcinomas (Verlaat et al., 2015). Thus, as a sequence of genetic alterations which occur in conjunction with distinct histopathological features in the development of HPV driven cervical squamous cell carcinomas, this permits the utilization of screening techniques for premalignant lesions.

Regarding the head and neck region, in 1996 Califano et al. published seminal research on the evolutionary genetic sequence of events for HNSCC, irrespective of their HPV status (Califano et al., 1996). The study documented genetic alterations and chromosomal changes that corresponded to histological findings, denoting a sequence of changes from normal mucosa to dysplasia, carcinoma *in-situ*, and finally invasive carcinoma. Loss of heterogenicity at 9p21, results in CDKN2A inactivation with subsequent transition from normal mucosa to hyperplasia (Johnson et al., 2020). This inciting event has been followed by mutation in TP53, a gene linked with DNA repair, with resultant histological evidence of transition from epithelial hyperplasia to dysplasia (Johnson et al., 2020). Furthermore, research to date has established that the genetic and chromosomal aberrations associated with cancer development occurs sequentially and are impactful based on their accumulative abnormalities rather than any individual mutation (Califano et al., 1996; Pai and Westra, 2009; Johnson et al., 2020).

However, the caveat and limitations of this model of tumorigenesis described is that it pertains to the head and neck region in general. It is not specific for the oropharynx, nor does it take into consideration the HPV status. CDKN2A and TP53 mutations are drivers of HPV positive cervical carcinoma, with their identification in the premalignant CIN lesions (Vassallo et al., 2000; Divya et al., 2017; Balasubramaniam et al., 2019). Thus, it could be postulated that such mutations identified may all be related to HPV driven tumorigenesis. However, both are described in HPV negative HNSCC, which will be discussed in further detail in later sections (Johnson et al., 2020).

Precursor lesions with an oncogenic potential have been identified within the head and neck region. These ‘white lesions’ are referred to as leukoplakias, with only 5–25% showing dysplasia, and an annual rate of transformation to a malignant lesion of approximately 2–3% (van der Waal, 2014; Neville, 2015). It is estimated that HPV detection in leukoplakias whether dysplastic or non-dysplastic varies from 0 to 50% (Miller and Johnstone, 2001; Campisi et al., 2007; Feller and Lemmer, 2012; Della Vella et al., 2020). The proliferative verrucous variant of oral leukoplakias is most commonly associated with HPV positivity, most predominantly the HPV 16 genotype (Feller and Lemmer, 2012). Although these premalignant lesions have a link to HPV, they are not identified within the oropharynx (Holmstrup et al., 2006).

In conclusion, there is no identifiable premalignant lesion directly resulting in OPSCC, in contrast to the cervix. However, HPV is deemed to have a role in the development of premalignant oral lesions. A lacking premalignant oropharyngeal lesion has limited the research prospective regarding the identification of distinctive somatic mutations or chromosomal abnormalities connected to the defined oncogenic pathway in HPV driven OPSCC.

### Human Papilloma Virus Genotype Within the Head and Neck Region

The predominant HPV genus linked with OPSCC is the alpha papillomavirus (de Villiers et al., 2004; Gheit, 2019). Further categorization of the alpha HPV genus is based on the oncogenic risk, extracted from work conducted predominantly on the anogenital region (LeConte et al., 2018). HPV genotypes are described as high risk if the prospect of developing a carcinoma was significantly more common than in individuals with HPV 6; which has been utilized as the pinacol for low risk (Cancero.t and o.C.R.t.H., 2012; Park et al., 2019). LR-HPV genotypes include HPV 6 and 11. HR- HPV types include 16, 18, 31, 33, 35, 39, 45, 51, 52, 56, 58, 59, and 68 (Muñoz et al., 2003; Cogliano et al., 2005; Kreimer et al., 2005; Nichols et al., 2013a; Haedicke and Iftner, 2013; Walline et al., 2013; Gheit, 2019; HSE, 2019).

Although the alpha genus is the most predominant genus linked with OPSCC, both the beta and gamma genera have been isolated from oral cavity samples (Bottalico et al., 2011; Garbuglia, 2014; Sabol et al., 2016; Wong et al., 2018). Studies have linked beta1 HPV 5, gamma 11 HPV and gamma 12 HPV with an increased risk of HNSCC, encompassing non-oropharyngeal regions of the head and neck (Bottalico et al., 2011; Agalliu et al., 2016; Sabol et al., 2016; Gheit, 2019). However, it has been extrapolated that the mechanism by which these particular non-alpha HPV genera propagate neoplastic development is comparable to the mechanism driving cutaneous HPV positive squamous cell carcinomas (Agalliu et al., 2016).

To date there is limited research concentrating on genotypes outside of the alpha genus in HNSCC and, in particular, OPSCC pathogenesis. The most prevalent HPV genotype identified in various studies of the head and neck region is HPV 16, accounting for 70–90% of cases (Kreimer et al., 2005; Evans and Powell, 2010; Dufour et al., 2012; Boscolo-Rizzo et al., 2013; Tumban, 2019; Kim et al., 2020). Within the oropharynx, the HPV 16 genotype accounts for 84–87.9% of cases (Goodman et al., 1990; Kreimer et al., 2005; Chaturvedi et al., 2008).

**FIGURE 2:**
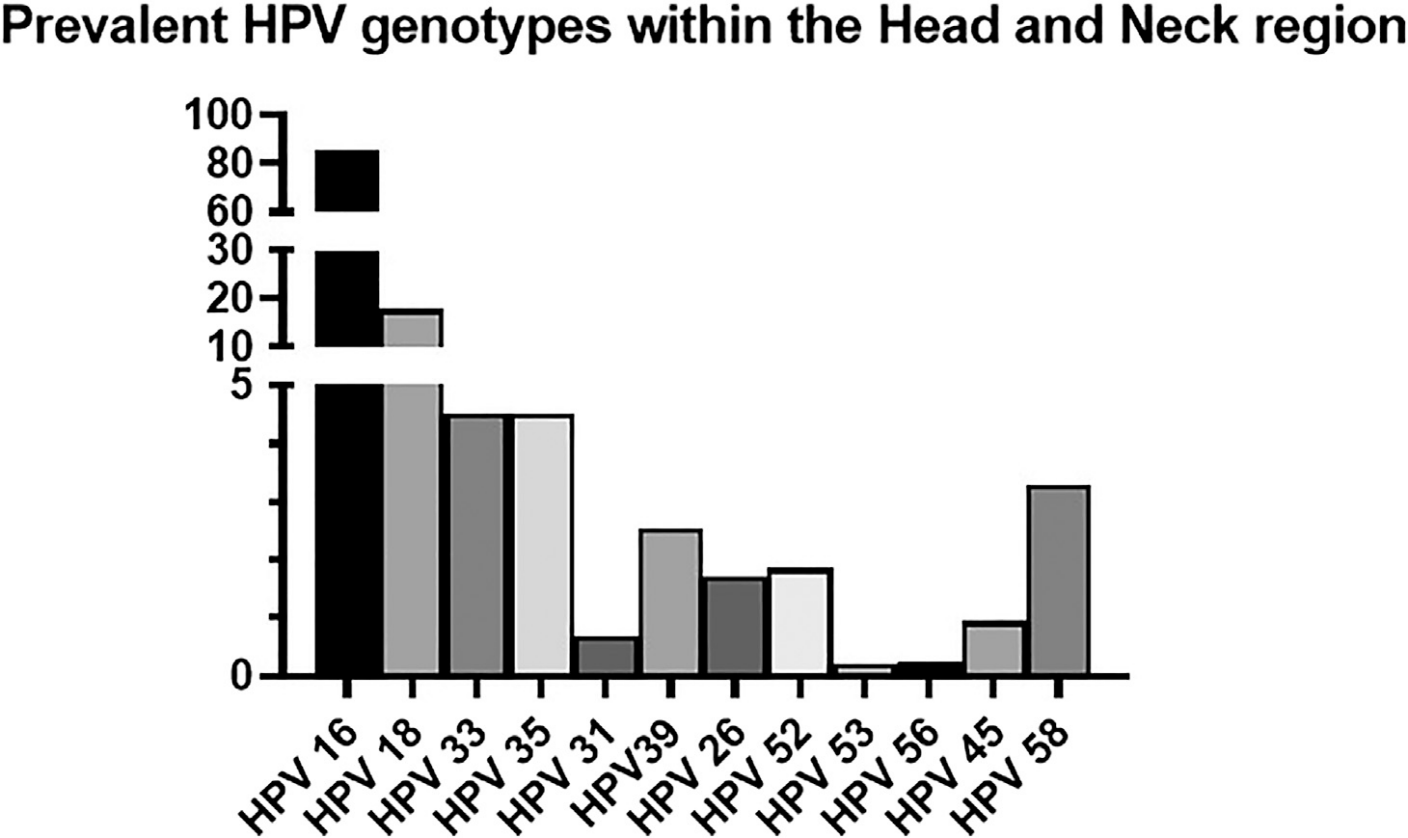
Prevalent HPV genotypes within the Head and Neck region (Goodman et al., 1990; Koskinen et al., 2003; Kreimer et al., 2005; Chaturvedi et al., 2008; Evans and Powell, 2010; Deng et al., 2012; Dufour et al., 2012; Boscolo-Rizzo et al., 2013; Ndiaye et al., 2014; Castellsagué et al., 2016; de Martel et al., 2017; Ursu et al., 2018; Gillison et al., 2019a; Tumban, 2019; Kim et al., 2020).

It is worth noting that not all studies discriminate findings relating solely to the oropharynx but combine figures from all regions of the head and neck. Within the head and neck region HPV 18 accounts for 14–21.7% (Ursu et al., 2018; Tumban, 2019). HPV 33 and HPV 35 both account for 4.5% (Kreimer et al., 2005; Ndiaye et al., 2014; Castellsagué et al., 2016; de Martel et al., 2017; Tumban, 2019). The less commonly identified genotypes are: HPV 31 (0.3–1.06%), HPV 39 (0.8–4.3%), HPV 26 (1.7%), HPV 52 (1–2.7%), HPV 53 (0.3%), HPV 56 (0.25%), HPV 45 (0.5–1.4%), HPV 58 (0.6–5.8%) (Goodman et al., 1990; Koskinen et al., 2003; Kreimer et al., 2005; Chaturvedi et al., 2008; Deng et al., 2012; Gillison et al., 2019a; Tumban, 2019). A full illustration of the prevalent HPV genotypes identified within the head and neck region is highlighted in Figure 2.

Comparing figures from the head and neck region to those in the cervix, a retrospective cross-sectional worldwide study reviewing HPV genotyping of invasive cervical carcinomas highlighted that HPV 16 accounted for 61% of cases, HPV 18 (10%), HPV 33 (4%), HPV 35 (2%), HPV 39 (2%), HPV 45 (6%), HPV 53 (<1%), HPV 56 (<1%) (de Sanjose et al., 2010). An interesting observation from the percentages outlined is that although HPV 16 is the most prevalent genotype in cervical and head and neck cancers, it contributes to a greater proportion of head and neck rather than cervical squamous cell carcinomas. The converse is that HPV 18 appears to have higher oncogenic potential in the cervix in contrast to the head and neck. No identifiable anatomical, physiological, or biochemical features have been elucidated as to why this is the case.

## Differences Between Human Papilloma Virus Positive and Negative Squamous Cell Carcinoma of the Head and Neck Region

### Genomic Characterization of Human Papilloma Virus Positive Versus Human Papilloma Virus Negative *Squamous Cell Carcinoma of the Head and Neck Region*

As noted earlier, research from the 1990s has provided instrumental knowledge regarding the carcinogenesis of HNSCC (Califano et al., 1996). The presence of mutations in TP53, CDKN2A, PIK3CA and Phosphatase and tensin homolog (PTEN) which are involved in the receptor tyrosine kinase/RAS/Phosphoinositide 3 kinase (RTK/RAS/PI3K) pathway; Notch homolog 1, translocation-associated (Drosophila) (NOTCH1) which is linked with squamous differentiation; (F-Box And WD Repeat Domain Containing 7) FBXW7 tumor suppressor gene which has a downstream effect on NOTCH1, were the initial mutations detected amongst HNSCC (Beck and Golemis, 2016; Litwin et al., 2017; Yeh et al., 2018; Gillison et al., 2019a; Huang et al., 2019). A seminal paper in 2000 by Gillison et al. established that HPV positive OPSCCs have a distinctive mutational landscape compared with HPV negative counterparts (Gillison et al., 2000). This study was the first to highlight the inverse relationship between TP53 mutation and HPV positivity (Gillison et al., 2000).

In 2011, two studies utilized whole genome sequencing to analyze the genetic framework of squamous cell carcinomas of the head and neck region (Agrawal et al., 2011; Stransky et al., 2011). These studies conducted by Stransky et al. and Agrawal et al. investigated not only OPSCC, but squamous cell carcinomas from other sites including the hypopharynx and oral cavity. Both studies documented that HPV positive squamous cell carcinomas from the head and neck lacked mutations in the TP53 gene (Agrawal et al., 2011; Stransky et al., 2011). These studies brought to the forefront the distinctive genetic aberrations between the HPV negative and positive cohort.

**FIGURE 3:**
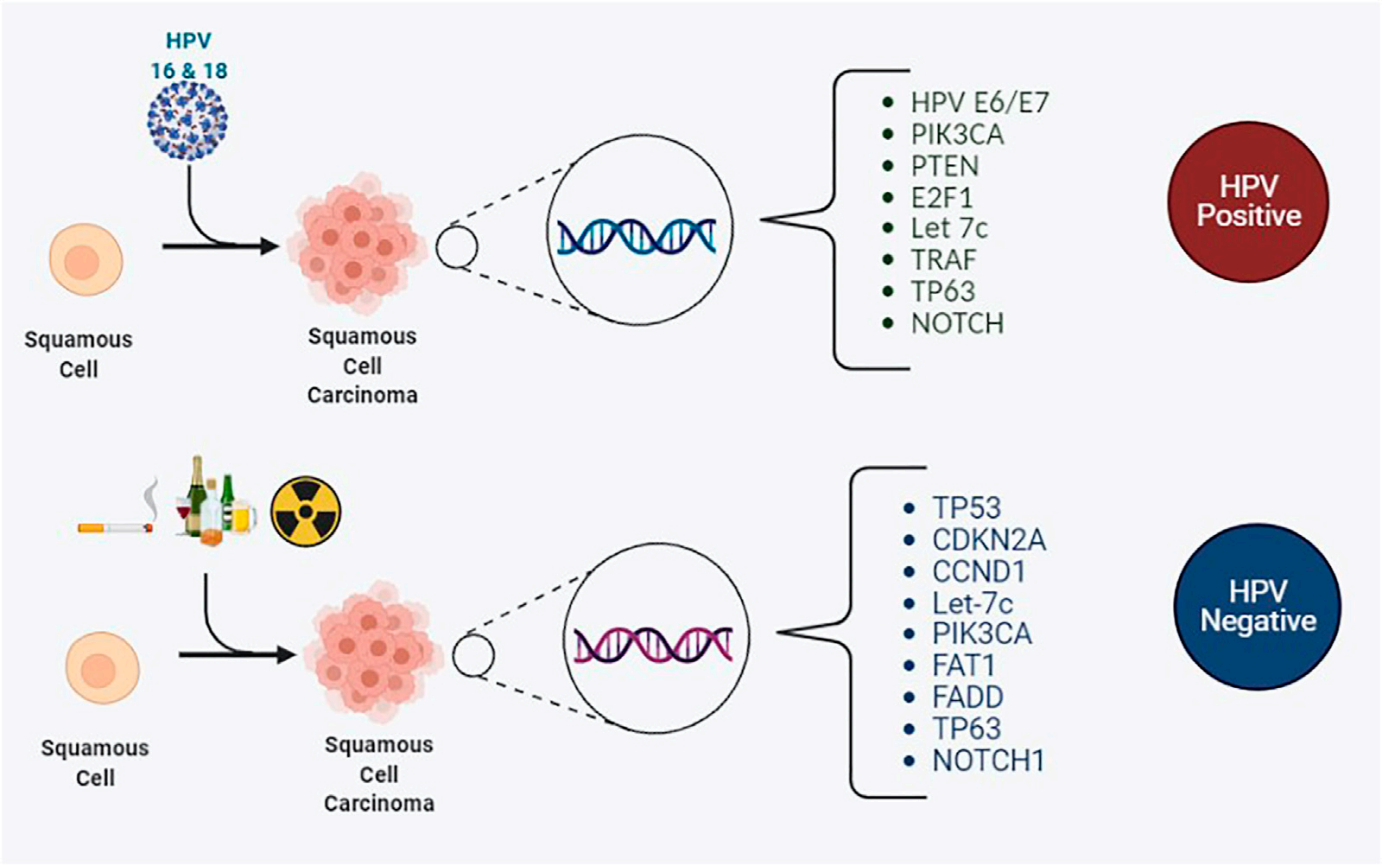
The most frequently encountered somatic mutations for HPV positive and HPV negative squamous cell carcinomas of the head and neck region.

In 2015 the Cancer Genome Atlas (TCGA) published its work underpinning the landscape of genomic alterations associated with HNSCC (Cancer Genome Atlas Network, 2015). In total, 279 HNSCC cases were analyzed in relation to DNA and RNA structural alterations, and somatic mutations, of which 36 cases of HPV positive HNSCC were identified within the cohort. The vast majority of the HPV positive cases arose from within the oropharynx, with the minority from within the oral cavity. A comprehensive list of mutations observed in HNSCC based on their HPV status from published literature to date is illustrated in Table 1 and Figure 3. The cited literature focuses on HPV positive HNSCC with figures derived from the oropharynx predominantly, with occasional references to other sites within the head and neck where HPV positivity was detected.

**TABLE 1:** Most frequent mutations identified in HPV positive and negative head and neck squamous cell carcinomas (Gillison et al., 2000; Agrawal et al., 2011; Stransky et al., 2011; Nichols et al., 2013c; Lechner et al., 2013; Pickering et al., 2013; Grønhøj Larsen et al., 2014; Cancer Genome Atlas Network, 2015; Rusan et al., 2015; Seiwert et al., 2015; Beck and Golemis, 2016; Chau et al., 2016; Marur and Forastiere, 2016; Hajek et al., 2017; Chen et al., 2018; Harbison et al., 2018).

HPV status	Gene	Percentage of cases
Positive	HPV E6/E7	**100**
—	PIK3CA	**10–56**
—	TRAF3	**5–28**
—	TP63	**11–28**
—	E2F1	**19**
—	Let 7c	**17**
—	NOTCH 1–3	**8–17**
—	PTEN	**6–15**
—	KMT2D	**13–14**
—	EP300	**10–12**
—	ZNF750	**11**
—	CYLD	**10**
—	RB1	**10**
—	HRAS, KRAS	**10**
Negative	TP53	**83–100**
—	CDKN2A	**25–58**
—	CCND1	**22–55**
—	Let-7c	**40**
—	PIK3CA	**13–34**
—	FAT1	**14–32**
—	FADD	**32**
—	TP63	**28**
—	NOTCH1	**13–26**
—	SYNE1	**22**
—	MUC16	**19**
—	USH2A	**18**
—	ZFHX4	**14–16**
—	MLL2	**16**
—	EGFR	**12–15**
—	MYC	**14**
—	LRP1B	**14**
—	NFE2L2	**14**
—	PIK3CB	**13**
—	URB5	**13**
—	PTEN	**12**
—	CASP8	**11**
—	FGFR1	**10**

The most frequently encountered mutated genes amongst HPV positive and negative OPSCC were TP53, CDKN2A, FAT1, PIK3CA, Lysine Methyltransferase 2D (KMT2D) and NOTCH1. These play a key role in the RTK/RAS/PI3K pathway. Other frequent mutations of OPSCC include let-7c and FGFR3, which are also linked to the RTK/RAS/PI3K pathway. Reviewing the Phosphoinositide 3 kinase pathway, genome analysis of OPSCC completed by next generation sequencing and copy number alterations displayed that regardless of HPV status and anatomic site, the somatic mutations are missense mutations on chromosome 3q26. 3q26 is the location of the PIK3CA gene (Davidson and Shanks, 2017). Phosphoinositide 3 kinases are a family of enzymes subdivided into three classes. They are activated by receptor tyrosine kinases and g-protein coupled receptors. The class I subgroup of the PI3K family is the most frequently associated with mutational dysregulation in HPV positive OPSCCs. Activation leads to phosphorylation of phosphatidylinositol 4,5-bisphosphate (PIP2) to phosphatidylinositol 3, 4,5-triphosphate (PIP3), which subsequently activates protein kinase B (AKT). AKT then proceeds to activate further targets downstream including mammalian target of rapamycin (mTOR). mTOR is divided into two complexes: Complex 1 and 2, which control biochemical and metabolic functions of the cell including lipid, protein and carbohydrate metabolism (Tan et al., 2019). The accumulative effect of this PI3K/AKT/mTOR pathway results in tumorigenic alterations in cellular functions including cell growth, differentiation, survival and migration (Psyrri et al., 2013; Isaacsson Velho et al., 2015). Furthermore, PTEN is frequently mutated in head and neck cancers, and its loss provides further amplification of the PI3K pathway (Psyrri et al., 2013). From mutational analysis, the PI3K/PTEN/AKT/mTOR pathway is the most frequently involved pathway involved in tumorigenesis of OPSCC (Chung et al., 2015). The fibroblast growth factor receptor 3 (FGFR3), is also linked with the PI3K pathway. FGFR3 permits signal transduction and subsequently activates mitogen‐activated protein kinase (MAPK), PI3K/AKT pathways (Koole et al., 2016).

The PI3K/PTEN/AKT/mTOR pathway has been identified as a key component of carcinogenesis in the HPV positive cohort in the literature to date. Given its prevalence in OPSCC, targeted agent against components of the pathway, including mTOR inhibitors, are being trialed in patients with advanced/metastatic disease irrespective of HPV status (Tan et al., 2019).

### Prognosis for Patients With Human Papilloma Virus Positive Oropharyngeal Squamous cell Carcinoma

HPV positive OPSCC are associated with young white males; a male to female ratio of 3:1; classically non-smokers; and it has been deemed to be a distinct clinical entity from HPV negative counterparts. Although HPV is a risk factor for the development of OPSCC, the overall prognostics are favorable in contrast to those who are negative (Ang et al., 2010; Economopoulou et al., 2020). From the literature published during the advent of derogation into two cohorts based on HPV status, HPV positive OPSCC showed a 60% reduction in risk of death from cancer, taking into account age, alcohol consumption and lymph node involvement (Gillison et al., 2000).

Subsequent studies have demonstrated increased sensitivity to treatment, with higher response rates to RT, induction chemotherapy and chemo-radiation therapy (CRT) (Marur et al., 2010; Bol and Grégoire, 2014). The two year progression free survival (PFS) rates for HPV positive and negative HNSCC range from 72 to 86%, and 50–75%, respectively (Marur et al., 2010). The two-year overall survival (OS) rates range from 88 to 94%, and 58–77%, respectively (Marur et al., 2010). The five-year survival from studies in Canada and Austria showing a greater divergence with figures of 83 and 85.7%, compared to 11.1 and 22.2% (HPV positive vs. negative) (Nichols et al., 2013b; Lill et al., 2017).

The following are some of the factors believed to contribute to the differences in outcome:

1) Given the characteristic younger age at diagnosis, individuals may have less co-morbidities, with a good baseline performance status prior to starting treatment (Ang et al., 2010; Elrefaey et al., 2014).
2) Studies have shown that HPV OPSCC are more radio-sensitive. Mechanisms by which this has been illustrated include the role of TP53 tumor suppressor gene and high oxygenation levels within HPV positive tumors in contrast to HPV negative tumors (Liu et al., 2018). HPV negative OPSCCs are associated with a mutated TP53 gene, which is critical for normal cell cycle regulation (Kastan, 2007). However, HPV positive OPSCC are shown to express the wild type p53 protein, thus maintaining a degree of normal functionality. RT induces cellular stress and damage occurring *via* p53 activating pro-apoptotic genes such as BCL2 Associated X, Apoptosis Regulator (Bax) (Liu et al., 2018). This is believed to play a key role in the favorable response rates of HPV positive OPSCC to RT (Kimple et al., 2013; Elrefaey et al., 2014; Liu et al., 2018).

A further theory pertains to the knowledge that HPV positive OPSCC are less hypoxic than HPV negative OPSCC. Oxygen is required for free radical formation, and thus the effectiveness of RT. In summation, higher oxygen and oxygen free radicals levels derived from HPV may thus be linked to overall improved radio-sensitivity (Liu et al., 2018).

3) Another proposed mechanism is *via* Immune regulation. Orthotopic models have shown immunocompetent mice infected with HPV positive cancer do better than those who are immunosuppressed (Nordfors et al., 2013; Turksma et al., 2013; Lechien et al., 2019). HPV OPSCC patients have a shift towards a greater number of tumor infiltrating lymphocytes such as CD8 positive T-Cells, CD8 positive T-cells who express PD1, and C4 positive T-cells (Nordfors et al., 2013; Turksma et al., 2013; Lechien et al., 2019). Studies have also shown that patients with HPV positive tumors have a greater number of effector memory T-cells in peripheral blood (Turksma et al., 2013). The proposition is that the immune response to tumor cells harboring HPV may provide a significant impact on anti-tumor progression.
4) Finally, differences in the mutational status of HPV positive vs. HPV negative OPSCC are believed to benefit the HPV positive cohort which will be discussed in detail below.

### Treatment Guidelines for Patients With Human Papilloma Virus Positive Oropharyngeal Squamous Cell Carcinoma

Despite outcomes varying, at present treatment guidelines for HPV positive disease are as per those for HPV negative (Marur and Forastiere, 2016; Machiels et al., 2020). In keeping with the National Comprehensive Cancer Network (NCCN) clinical practice guidelines in Oncology for Head and Neck cancers and the European Head and Neck Society, the European Society for Medical Oncology, and the European Society for Radiotherapy and Oncology (EHNS-ESMO-ESTRO) clinical practice guidelines, HPV detection should not change treatment plans for the patient (Head and Cancers, 2017; Kim et al., 2018; Machiels et al., 2020). The standard therapy for newly diagnosed HNSCC is dependent on the TNM stage and ranges from RT, chemoradiotherapy (CRT) and/or surgical intervention (Machiels et al., 2020).

Curative therapies, although effective in their aim to improve PFS and OS, are not without their risks and complications. The toxicity associated with chemotherapeutics and RT can be extremely challenging and debilitating for patients, thus the importance of providing an adequate dosage of a therapeutic agent without inducing localized and/or widespread complications. This is extremely important given the younger cohort of patients with HPV positive disease, who potentially will live longer enduring side effects including xerostomia, dysphagia and the requirement for parenteral feeding tubes (Wirth et al., 2019). It is known that HPV positive OPSCC are more sensitive to chemotherapy, RT and combined CRT (Liu et al., 2018; Economopoulou et al., 2019). This has resulted in the generation of the field of research analyzing strategies of de-intensifying therapies for HPV positive OPSCC to reduce treatment induced morbidity. Mechanisms investigated include dose reduction or elimination of chemotherapy, implementing alternative systemic therapies, dose reduction and field reduction of RT, and/or alternative surgical techniques (De Felice et al., 2019).

Intensity modulated RT involves administration of a reduced treatment dose, with studies administering fractions ranging from 30 to 60 Gy (Chen et al., 2017; Nevens et al., 2017; Tsai et al., 2019; Yom et al., 2019). Preliminary results from phase II and III of trials show no inferiority in maintaining loco-regional control and PFS. However, the follow up time frame is limited, with no long term follow-up studies published (Chen et al., 2017; Nevens et al., 2017; Yom et al., 2019). A further mechanistic strategy is that of targeted reduced dosage of RT, with research ongoing in Memorial Sloan Kettering regarding delivery of a standard 70 Gy to visible disease, with delivery of 30 Gy to elective regions (sites with no gross identifiable disease) (Tsai et al., 2019). Of note, there is no provision within the EHNS-ESMO-ESTRO guidelines for amendments to the set protocol guidelines for radiation dose reduction for HPV positive OPSCC given the prematurity of this data (Machiels et al., 2020).

As highlighted above, mechanisms to reduce toxicity and subsequently improve quality of life relate to administration of an alternative agent to the mainstay systemic chemotherapeutic agents. Such agents utilized include Cisplatin, or a platinum therapy combined with 5-Fluorouracil. One of the proposed substitutes involves targeting receptors linked to pro-oncogenic activity in conjunction or as an alternative to systemic chemotherapy. Molecular analysis of OPSCC patients identified high levels of epidermal growth factor receptor (EGFR) expression, predominantly in the HPV negative cohort, as illustrated in Table 1 (Bonner et al., 2010; Rehmani and Issaeva, 2020). This high expression has been linked to reduced OS, reduced radio-sensitivity and increased recurrence post RT (Ang et al., 2002; Bonner et al., 2010; Tejani et al., 2010). EGFR, a transmembrane receptor, is a member of the erbB category of type I receptor tyrosine kinases with downstream signaling involving the microtubule associated protein kinase/eukaryotic protein kinase (MAPK/EPK), Janus Kinase/Signal Transducer and Activator of Transcription (JAK/STAT) and PI3K/AKT pathways. These pathways all have implications on cell proliferation, cell survival, evasion of apoptosis, angiogenesis and cell migration (Tejani et al., 2010; Falzone et al., 2018). The dysregulation of EGFR within this cohort of patients has resulted in the utilization of Cetuximab, an Immunoglobulin G1 (IgG1) targeted monoclonal antibody, that targets EGFR.

EGFR targeted therapies for HNSCC have shown to improve OS benefit when administered with RT, with a landmark study showing such in 2000 by Bonner et al. Bonner et al. (2000); Bonner et al., 2010). In this study, patients were included irrespective of EGFR status. The findings of this study promoted a new focus of research with additional studies investigating Cetuximab in HPV positive OPSCC. The De-ESCAlaTE trial replaced Cisplatin with Cetuximab with concomitant RT for patients with HPV positive OPSCC. This study showed no benefit for toxicity, however a reduction in tumor control was observed (Mehanna et al., 2019; Oosthuizen and Doody, 2019). A further study by Gillison et al. showed that RT plus cetuximab demonstrated inferior OS and PFS compared to RT plus Cisplatin, with similar toxicity rates (Gillison et al., 2019b; Oosthuizen and Doody, 2019). In summary, Cetuximab has not been shown as an alternative for systemic chemotherapy and the current treatment guidelines issued do not endorse the usage in replacement of systemic chemotherapy in treating HPV positive OPSCC (Machiels et al., 2020).

Further de-intensification measures such as replacing CRT with RT alone have been trialed. A retrospective study from 2013 showed a comparable outcome for HPV positive OPSCC treated with RT alone in contrast to those treated with CRT (Chen et al., 2013; De Felice et al., 2019). However, preliminary results from a phase II trial reviewing intensity modulated RT with chemotherapy vs. modulated RT alone, show the 2 years PFS of 90.5% for combined therapy vs. 87.6% for RT alone (Yom et al., 2019).

Additional studies are reviewing various modalities to reduce intensity and thus toxicity of therapy without compromising cancer control. An example include a Phase II trial examining administration of induction Cisplatin chemotherapy, followed by Cetuximab with reduced dose RT for HPV-positive resectable stage III/IV OPSCC (Marur et al., 2017). The study showed reduced delayed swallowing issues for those who received induction chemotherapy and reduced dose RT and recommended further large-scale studies. The Quarterback trial, a Phase III trial, examined the effects of a reduced dose CRT after induction chemotherapy vs. standard CRT. The result showed similar PFS and and OS in both groups (Misiukiewicz et al., 2019).

The utilization of ‘organ preservation surgery’ to ensure minimal long term morbidity is an increasing are of interest acknowledged in current guidelines (Machiels et al., 2020). Results from a Phase II trial in 2020 utilizing transoral robotic surgery with intensity modulated RT has shown promising results in HPV positive OPSCC (Ferris et al., 2020). Additional trials reviewing less invasive surgical techniques include PATHOS (primary surgical de-escalation trial in Post-Operative Adjuvant Treatment of HPV-Positive Tumors) and ADEPT (Adjuvant De-escalation, Extracapsular Spread, P16 Positive, Transoral trial) are ongoing (Trials, 1687; Trials, 2014).

## Human Papilloma Virus Positive Oropharyngeal Squamous Cell Carcinoma Linked With Disease Progression/Recurrence

### Human Papilloma Virus Genotypes Linked With Disease Progression/Recurrence

Despite the definitive association of HPV positivity with overall better outcomes in OPSCC, there is limited research to date studying the impact of each HPV genotype with respect to OS. This may be as the HPV 16 genotype is the most prevalent genotype identified OPSCC (Figure 2). Lei et al. highlighted that HPV 18 is associated with a worse prognosis than HPV 16 in invasive cervical carcinoma (Lei et al., 2019). A review of nearly 700 cases of HPV positive invasive cervical cancer from an American cancer registry showed an improved 5 years survival in HPV 31/33/44/52/58 subtypes in contrast to HPV 16 and HPV 18 (Hallowell et al., 2018).

Regarding the head and neck region, a multicenter study in Korea published in 2015 revealed that tonsillar carcinomas which were positive for the HPV 18 genotype were associated with shorter disease-free intervals than that of the HPV‐negative and HPV 16 positive groups (No et al., 2015). Data from TCGA conflicted with the Korean study, in that HPV positive OPSCC subtypes other than HPV 16 had a more favorable OS (Cancer Genome Atlas Network, 2015; Beck and Golemis, 2016; Bratman et al., 2016). It is worth noting that HPV 18 was not detected in this cohort. Thus, correlating particular genotypes with PFS or OS may be an area worth exploring in the future.

### Genetic Aberrations Linked With Disease Progression/Recurrence

To date there is limited data examining the genetic aberrations associated in those with HPV positive OPSCC who recur (Tinhofer et al., 2016a; Tinhofer et al., 2016b; Harbison et al., 2018; Reder et al., 2019). Two key studies by Reder et al. and Harbison et al. collectively analyze 28 patients with documented recurrences (Harbison et al., 2018; Reder et al., 2019) The working hypothesis was that HPV positive OPSCC that recur share a mutational landscape similar/in keeping with HPV negative OSPCC, thus explaining the poor prognosis (Harbison et al., 2018; Reder et al., 2019).

Combining the two studies of the HPV positive OPSCC, the most common mutations identified amongst the HPV positive OPSCC groups that did and did not recur included: TP53, RB1, KMT2D, PIK3CA, TAF1, CYLD, EP300, PEG3, STAT3, BCL6, FBXW7, NCOR1, NSD1, DDX3X, PDGFRA, NOTCH1 (Reder et al., 2019; Harbison et al., 2018). Mutations of TSC2, BRIP1, NBN, TACC3, NFE2L2, STK11, HRAS, PIK3R1, TP63 and FAT1 were isolated to the recurrence group only (Table 2)^.^ FGFR3, B2M, TRAF3, SMAD2, ARID, MAPK1 and MAPK2K2 mutations were observed in those who did not recur (Harbison et al., 2018; Reder et al., 2019).

**TABLE 2:** Most frequent mutations identified in patients with HPV positive OPSCC who developed recurrence and/or metastasis.

HPV positive OPSCC	Mutations detected in recurrence group
—	TSC2
—	BRIP1
—	NBN
—	TACC3
—	NGE2L2
—	STK11
—	HRAS
—	PIK3R1
—	TP63
—	FAT 1

Regarding the mutations identified solely in the recurrence group, PIK3R1, TSC2 and STK11 (LKB1) are involved in the PI3K/AKT/mTOR pathway. Tuberous Sclerosis Complex subunit 2 (TSC2) is a tumor suppressor gene which produces the protein tuberin. This protein interacts with the hamartin protein produced by Tuberous Sclerosis Complex subunit 1 (TSC1) (Huang and Manning, 2008). Hamartin-tuberin complex exhibit a GTPase activating activity on RHEB (Ras homolog enriched in brain). The downstream effect is the inhibition of mTOR, thus the connection to the PI3K/AKT/mTOR pathway (Manning and Cantley, 2003; Huang and Manning, 2008). Mutated TSC2 results in upregulation of this pathway. The tumor suppressor gene serine threonine kinase 11 (STK11), also referred to as liver kinase B1 (LKB1), activates adenosine monophosphate-activated protein kinase (AMPK) which has an inhibitory effect on mTOR. Mutation of STK11 leads to the upregulation of the mTOR pathway (Iglesias-Bartolome et al., 2013; Zhou et al., 2013).

TP63 is a tumor suppressor gene from the same family of a TP53. The TP63 gene is expressed in numerous isoforms, most relevant thus far to tumorigenesis are TAp63 and ΔNp63(Bergholz and Xiao, 2012). TP63 has been linked to cell survival, senescence, cellular differentiation and apoptosis (Bergholz and Xiao, 2012). The loss of function of the TP63 gene, can result in the activation of MAPK. This can promote the upregulation of the Mitogen Associated Protein Kinase - Signal Transducer And Activator Of Transcription 3-Matrix Metalloproteinase five axis, which has been shown to promote metastasis (Lakshmanachetty et al., 2019).

The next gene mutation linked with HPV positive OPSCC who recur is the BRIP1(BRCA1 interacting protein C-Terminal Helicase 1). This gene is associated with Fanconi’s anemia, a condition linked to HNSCC(O'Donnell et al., 2018). NBN gene (also known by NBS1) produces protein nibrin, which plays an instrumental role in DNA repair (O'Donnell et al., 2018; Daino et al., 2013; Desjardins et al., 2009).

A further mutation discovered includes a mutation in transforming acidic coiled coil containing protein 3 (TACC3) gene. This gene produces a non-motor protein that functions to promote microtubule growth from the centrosome, thus providing stability to the mitotic spindle. This occurs *via* a transforming acidic coiled-coil protein 3 (TACC3)/colonic, hepatic tumor overexpressed gene (ch-TOG)/clathrin complex (Booth et al., 2011; Ding et al., 2017).

Additional mutation of nuclear factor–erythroid-2–related factor-2 (NFE2L2), a gene that encodes the transcription factor NF-E2-Related Factor 2 (Nrf2). Nrf2 plays a role in the antioxidant response within the cell, with mutation leading to resistance of the cell to anti-oxidant damage and ensuring cell survival (Qian et al., 2015; Gilfillan et al., 2019). FAT1 gene is a member of the cadherin family. It is a homologue of Drosophila tumor suppressor gene fat, and is involved in cell movement and inhibition of cell growth (Katoh, 2012).

Harvey rat sarcoma viral oncogene homolog is a member of the RAS family of oncogenes frequently mutated in a variety of cancers. The family is subdivided into HRAS, KRAS and NRAS. The RAS family of oncogenes are extremely important in tumorigenesis owing to their key role in signal transduction pathways. The RAS protein alternates between being bound to a GTP or GDP, with GDP being the inactive state. RAS signaling plays a key function for cellular aspects of cellular proliferation, survival, angiogenesis and differentiation (Muñoz-Maldonado et al., 2019).

There have been studies conducted to elicit whether particular somatic mutations are identified in this cohort of patients, in an attempt to correlate which patients are associated with a worse overall outcome. NOTCH1 and PTEN mutations were linked with a reduced recurrence free survival in patients with HPV positive OPSCC (de Carvalho et al., 2020), with PIK3CA mutations linked with reduced disease free survival in those on de-intensified CRT (Beaty et al., 2019). Conversely, FGFR3 mutations in HPV positive OPSCC are linked with improved disease free survival (BERSANI et al., 2018). A further study highlighted that FGPR3 mutations were predominantly identified in an HPV negative OPSCC cohort, and in combination with mutant TP53 was linked with a worse overall prognosis (Nannapaneni et al., 2021). Thus, extrapolating this data, combined mutant TP53 and mutated FGFR3 in the HPV positive OPSCC cohort is linked to improved OS.

There a two principal parting points regarding genetic aberrations in OPSCC tumors associated with disease progression and/or recurrence. Firstly, although hypothesized that the recurrence group have genetic mutations in line with the HPV negative cohort, Harbison et al. concluded that the HPV related OPSCC that did or did not recur had more genomic features in common with each other than with HPV-unrelated tumors (Harbison et al., 2018). Secondly, the caveat from the literature cited within this section is that the results depicted are all derived from studies each with a small cohort of patients, thus caution is warranted regarding interpretation. However, this has set the stage for ongoing innovative research in this area to assess what/if any mutational features may thus be associated with recurrence, which may provide in-depth future knowledge for targeted therapies.

### Treatment Guidelines for Patients With Human Papilloma Virus Positive Head and Neck Squamous Cell Carcinoma With Recurrence And/Or Metastasis

In patients with recurrent and/or metastatic disease, the mainstay of treatment is dependent on numerous factors including site of recurrence, resectability and prior treatment (Machiels et al., 2020). Treatment regimens can vary from surgical intervention, RT, systemic chemotherapy and utilization of novel agents including immune check point inhibitors (De Felice et al., 2019; Machiels et al., 2020).

Cancer development results in cancer cells being capable of evading immune response to ensure their survival. Non-cancer cells possess immune-checkpoint proteins, which act as a protective mechanism, preventing an autoimmune attack on the host. In cancer evolution, neoplastic cells possess such checkpoint proteins, and thus evade the host immune system (Ganesan and Mehnert, 2020). Targeting the immune checkpoints prevents evasion of the immune response to the cancer. The main categories include cytotoxic T lymphocyte associated antigen four antibody (CTLA-4) and PD1/PDL1 (McCusker et al., 2020). PD1 is expressed by CD8 positive cytotoxic T lymphocytes (Alsaab et al., 2017; Ganesan and Mehnert, 2020). PDL1, expressed by tumor cells, interacts with PD1 resulting in suppression of T lymphocyte activity. Anti-PDL1 therapies developed, such as Pembrolizumab, target PDL1 thus leaving PD1 unopposed and permitting activity of CD8 positive T lymphocytes. Current treatment guidelines advise their utilization be reserved for recurrent and/or metastatic disease irrespective of HPV status (Machiels et al., 2020).

Lyford-Park et al., in 2013 published a central paper demonstrating the role of PD1/PDL1 in providing an environment for HPV infection and persistence, with provision to eliminate the immune regulation and resultant tumorigenesis (Lyford-Pike et al., 2013a). Studies have illustrated a strong correlation between HPV positivity and PD1/PDL1 expression (Hong et al., 2016; Balermpas et al., 2017; Yang et al., 2018; Hong et al., 2019). PDL-1 expression has been linked to improved outcomes in certain studies (Hong et al., 2016; Hong et al., 2019; Patel et al., 2020). However a recently published systemic review and meta-analysis, showed PDL1 expression was linked with improved OS, with HPV status showing no impact on the findings (Patel et al., 2020).

Nivolumab, an IgG4 anti–PD1 monoclonal antibody, has been trialed for recurrent HNSCC, irrespective of HPV status, and has shown improved OS in contrast to those on single agent Docetaxel, Methotrexate or Cetuximab (Ferris et al., 2016). Although this study looked at all head and neck cancers, the HPV positive cases had displayed longer duration of OS (Forster and Devlin, 2018). Thus, this may be an area for clinical studies in the future. The KEYNOTE-048 study proved that combination of standard chemotherapy with Pembrolizumab significantly improved OS than chemotherapy alone for recurrent or metastatic disease (Burtness et al., 2019). This study does not take into account the HPV status.

A further phase II trial, HAWK, examined the utilization of Durvalumab monotherapy vs. systemic chemotherapy in patients with recurrent or metastatic HNSCC that had high PDL1 expression in tumor cells. When stratified based on site and HPV status, 30% of patients with HPV-positive OPSCC had a response in contrast to 10.8% in the HPV negative cohort (Cohen et al., 2019; Zandberg et al., 2019).

### Human Papilloma Virus Clearance Post Treatment–Current Concepts

One area that has been studied in recent times involves the prospect of detecting HPV DNA in the oral cavity by oral rinses or swabs, or *via* hematological confirmation by circulating free DNA (cfDNA) detection in plasma (Gipson et al., 2018). Such measures have been implemented in studies as a tool to assess the response to treatment with the conceivable prospect that the oncologic virus if eliminated by treatment would correlate with a reduced risk of recurrence and/or metastasis.

The utilization of viral DNA detection as a surrogate marker for treatment response within the head and neck region is not a new entity. The Epstein-Barr Virus is a known oncogenic virus linked to the development of nasopharyngeal carcinoma (Hau et al., 2020). In 1998 Mutirangura et al. explored the detection of circulating EBV DNA as a biomarker for nasopharyngeal carcinoma utilizing conventional PCR (Mutirangura et al., 1998). In 1999, Lo et al. took it one step further by utilizing real time PCR to identify circulating free EBV DNA (Lo et al., 1999; Chan, 2014). Over time, quantification measures were introduced by Lo et al. and EBV DNA expression level may be used as a promising prognostic factor to predict the outcomes in nasopharyngeal carcinoma (Zhang et al., 2016). This measure was a pivotal process within the management of head and neck cancers, as it was the first time within the head and neck region that analysis of an oncogenic virus was utilized as a mechanism to steer patient management (Chan, 2014).

A systematic review of eight studies found the sensitivity and specificity for HPV detection based on oral rinse or an oral swab ranged from 72 to 92% respectively (Gipson et al., 2018). Conversely, the sensitivity for the detection of HPV circulating tumor DNA within the plasma is much lower. The published literature states that the sensitivity for detection is extremely variable, with rates ranging from 19 to 79% (Gipson et al., 2018). However, progress within this area has been highlighted recently. In 2020 Chera et al. demonstrated a sensitivity rate of 100% and a specificity rate of 99% (Chera et al., 2019).

An interesting perspective from HPV detection *via* the methods outlined is the potential to assess if viral clearance has been achieved post cancer treatment. A study by Fahkry et al. published in 2019 reviewed HPV status from oral rinses post treatment regimens for patients with oral and oropharyngeal cancers and highlighted the percentage of patients who never achieve full clearance of their HPV (Fakhry et al., 2019). For individuals who underwent surgical resection, the percentage of those who had HPV DNA detected in oral rinses dropped from 69.2 to 13.7%. For those who underwent surgical excision and adjuvant RT, percentages dropped from 70 to 1%. The final category of patients who underwent RT with or without chemotherapy, HPV detection dropped from 85 to 9%. Reviewing the utilization of circulating free HPV DNA (cfHPVDNA) within the blood plasma post treatment, a recent study highlighted that cfHPVDNA was detected in 76% of patients (Chera et al., 2019). Thus, despite optimal management *via* various therapeutic modalities, HPV clearance is not always achieved.

Translating such findings into the clinical domain, Fahkry et al. showed that patients with cfHPVDNA post treatment have a 2-years incidence of recurrence without detectable tumor HPV DNA of 45.3%, in contrast to 12.2%. This was linked to an increased risk of local and regional recurrence (Fakhry et al., 2019). Ahn et al. found that HPV-positive OPSCC who have HPV 16 DNA detected in salivary samples post treatment were associated with a higher risk of recurrence and death (Ahn et al., 2014). Further research utilizing combined salivary and hematological tests showed that the presence of HPV in both testing mechanisms was linked to an increased risk of recurrence (Ahn et al., 2014).

In relation to the particular genotype strains detected, certain studies reviewed only HPV 16 DNA post-treatment, whereas Ahn et al. reviewed numerous HR and LR HPV subtypes (Ahn et al., 2014; Chera et al., 2019; Fakhry et al., 2019). As expected, HPV 16 being the most prominent genotype detected (Ahn et al., 2014). HPV 58 and HPV 35 displayed the highest viral load reference figures at baseline, and accounted for 1.5 and 1.9% of cases (Ahn et al., 2014; Fakhry et al., 2019). Of note, HPV 16 was the most prevalent genotype detected in the final sample, highlighting its persistence. However, a single sample detected the HPV 51 genotype, and interestingly the viral load at the final sample was greater than the initial viral load detected on first sample (Ahn et al., 2014; Fakhry et al., 2019).

In summary, HPV detection by routine procedures such as oral rinses or blood analysis in patients who have undergone treatment may in the future be utilized as a screening mechanism to stratify patients into those who are higher risk of recurrence based on their inability to clear the oncogenic virus.

## Conclusion

HPV driven cancers within the oropharynx have marked pathogenic differences in contrast to HPV driven cervical cancers. HPV positive status proffers a favorable prognosis for HPV positive OPSCC in contrast to its HPV negative counterparts. However, cases of HPV positive OPSCC recurrence do occur, and are linked with poor poorer overall outcomes. There is limited research to date examining the genotypic strains and the mutational landscape of HPV positive OPSCC patients who experience local and/or distant recurrences. With ongoing research into de-intensifying therapeutic regimens for HPV positive OPSCC, the identification of those individuals likely to progress or re-present with recurrence or metastasis is paramount. Concentrating on genotypic and/or mutational patterns in the recurrence group, as well as identification of individuals with residual virus within the oral cavity post cancer treatment, may result in improved stratification of treatment for patients with HPV positive OPSCC.
